# Anæsthetics: Their Uses and Administration

**Published:** 1888-12

**Authors:** 


*Anæsthetics: their Uses and Administration.* By DUDLEY WILMOT BUXTON, M.D. Pp. 164. London: H. K. LEWIS. 1888.

We have formed a most favourable opinion of this little work, the latest of Lewis's Practical Series. Written by a man of large experience, who has also studied the literature of his subject, the book is just what one seeking to become an administrator of anæsthetics wants. It is eminently practical; well up to date; clearly and tersely written; and is very complete. We may congratulate the author on having succeeded in his attempt "to indicate that the matter dealt with has a scientific as well as a work-a-day aspect, and that he who desires to be more than a mechanical (and hence dangerous) administrator of anæsthetics, must be scientifically as well as practically educated in his art."

